# Molecular Cloning and Functional Characterization of Heat Stress-Responsive Superoxide Dismutases in Garlic (*Allium sativum* L.)

**DOI:** 10.3390/antiox10050815

**Published:** 2021-05-20

**Authors:** Hyo Seong Ji, Seoung Gun Bang, Min-A Ahn, Gayeon Kim, Eunhui Kim, Seung Hee Eom, Tae Kyung Hyun

**Affiliations:** Department of Industrial Plant Science and Technology, College of Agricultural, Life and Environmental Sciences, Chungbuk National University, Cheongju 28644, Korea; wlgytjd007@chungbuk.ac.kr (H.S.J.); qkdtjdrjs777@chungbuk.ac.kr (S.G.B.); koala0523@chungbuk.ac.kr (M.-A.A.); gayeon0508@chungbuk.ac.kr (G.K.); rupinus99@chungbuk.ac.kr (E.K.); eom0214@naver.com (S.H.E.)

**Keywords:** antioxidant, garlic, heat stress, superoxide dismutase

## Abstract

Superoxide dismutases (SODs) are key antioxidant enzymes that can detoxify the superoxide radicals generated by various stresses. Although various plant SODs have been suggested to improve stress tolerance, SODs in garlic, an economically important vegetable grown worldwide, remain relatively unknown. In this study, we found that heat stress strongly induced the activities of Cu/ZnSODs, FeSODs, and MnSODs in garlic leaves. In addition, we cloned four garlic *SODs* (*AsSODs*) and suggest that heat stress-increased SOD activity was reflected at least by the induction of these *AsSODs*. The results of the agro-infiltration assay suggested that the cloned *AsSODs* encoded functional SOD enzymes belonging to the Cu/ZnSOD and MnSOD families. As a first step toward understanding the enzymatic antioxidant system in garlic plants, our results provide a solid foundation for an in-depth analysis of the physiological functions of the AsSOD family.

## 1. Introduction

In plants, reactive oxygen species (ROS) are regarded as byproducts of various redox reactions in several cellular compartments, including chloroplasts, mitochondria, peroxisomes, apoplast, and plasma membranes [1,2]. Although ROS are an unavoidable byproduct of aerobic metabolism, the most common effect of environmental stresses such as drought, salt, and extreme temperature is the generation of ROS, including free radicals (e.g., superoxide radicals O_2_^•−^ and hydroxyl radical ^•^OH) and non-radical oxidants (e.g., hydrogen peroxide H_2_O_2_). In general, optimum levels of ROS are required for cell function, as they are important signaling molecules that regulate growth, development, and response to various stresses [3]. However, excessive ROS production due to environmental stresses results in damage to cellular components such as carbohydrates, proteins, lipids, and DNA and eventually leads to metabolic disorder and cell death in plants [1]. Thus, ROS acts as a double-edged sword, and the balance between ROS and the antioxidant systems is highly critical in maintaining cellular health [2].

Plant antioxidant systems can be classified as enzymatic and non-enzymatic. Among them, superoxide dismutases (SODs), which are ubiquitous metalloenzymes, play a major role in the first line of antioxidant defense by catalyzing the dismutation of superoxide radicals to H_2_O_2_ and O_2_ [4]. On the basis of the type of metal cofactor at the active site of the enzyme, plant SODs are classified as copper/zinc SODs (Cu/ZnSODs), iron SODs (FeSODs), and manganese SODs (MnSODs) [4]. Because of their essential role in antioxidant systems, various SOD genes have been widely studied in many plant species, including Arabidopsis [5], grapevine [6], rapeseed-mustard crops (*Brassica juncea* and *B. rapa*) [7], tea [8], wheat [9], *Salvia miltiorrhiza* [10], barley [11], soybean [12], and *Zostera marina* [4]. In rice, overexpression of *OsCu/ZnSODs* can improve ROS detoxification capacity, resulting in enhanced resistance against saline–sodic stress [13]. Similarly, overexpression of *SODs* in various plants confers tolerance to abiotic stresses, including oxidative, drought, and salt stress [14,15,16]. Increased temperature leads to the production of superoxide radicals and H_2_O_2_, which causes heat stress-induced oxidative damage in plants [17]. In rice, overexpression of Golgi/plastid-type rice *MnSOD1* improves the quality of rice grains produced under heat stress during ripening, indicating a positive correlation between higher SOD activity/expression and stress tolerance. Therefore, the total activity of SODs and the pattern of SOD isoforms have been used as biochemical markers for screening crop germplasm for abiotic stress tolerance [18,19].

Garlic (*Allium sativum* L.) has been cultivated globally for more than 5000 years as a vegetable, spice, and medicinal crop [20]. Despite the remarkable economic importance of garlic, identification and functional characterization of the antioxidant system in garlic, including garlic SODs, have not been performed in detail. In this study, we cloned four garlic *SODs* (*AsSODs*) belonging to the Cu/ZnSOD and MnSOD families. We also analyzed the expression pattern of these *AsSODs* in various tissues of garlic and in response to heat stress. Our results provide the foundation for further functional analysis of AsSODs and can be of great importance for obtaining gene resources to improve stress tolerance in garlic.

## 2. Materials and Methods

### 2.1. Plant Growth and Treatment

Seed cloves of Southern-type garlic (*A. sativum* L. cv. Daeseo), which is widely cultivated in the Republic of Korea, were obtained from Chungcheongbuk-do Agricultural Research & Extension Services, Garlic Research Institute, Republic of Korea, and grown in a growth chamber (24 °C; relative humidity, 50%; 16 h/8 h light/dark cycles). For heat treatment, 4-week-old plants were incubated at 35 °C or 45 °C for various durations (2 h, 5 h, and 8 h). The experiment was conducted with five biological replicates.

### 2.2. Histochemical Analysis of H_2_O_2_ Accumulation and Determination of Lipid Peroxidation

The accumulation of H_2_O_2_ in heat-treated leaves was detected by endogenous peroxidase-dependent in situ histochemical staining as described by Daudi and O’Brien [21]. Leaves from heat-treated garlic plants were vacuum-infiltrated with DAB solution containing 0.1% DAB, 0.05% Tween-20, and 200 mM Na_2_HPO_4_. The infiltrated leaves were incubated for 12 h under dark conditions, and their chlorophyll was removed by incubation with a bleaching solution (ethanol/acetic acid/glycerol = 3:1:1) at 90 °C. 

The cellular damage caused by heat-induced ROS was determined by analyzing the level of lipid peroxidation in garlic plants. Lipid peroxidation was detected by measuring the malondialdehyde (MDA) content as described by Eom and Hyun [22]. The MDA content (nmol /mg of fresh weight) was determined using the extinction coefficient (155 mM^−1^ cm^−1^).

### 2.3. In-Gel SOD Activity Assay and Analysis of Total SOD Activity

Total protein was extracted using an extraction buffer (0.2 M potassium phosphate buffer [pH 7.8], 0.1 mM EDTA, and 1% protease inhibitor cocktail). Total protein content was determined using the Pierce™ BCA Protein Assay Kit (Thermo Fisher Scientific, Rockford, IL, USA). Total SOD activity was analyzed using the NBT method as described by Beyer and Fridovich [23].

The in-gel SOD activity assay was performed as described [16], with minor modifications. We separated 50 µg of total protein by native-PAGE on 10% polyacrylamide gels, and the gels were then incubated with a reaction mixture (0.48 mM nitroblue tetrazolium, and 30 µM riboflavin in 50 mM K-phosphate buffer, pH 7.8) for 30 min in dark conditions. The gels were illuminated until white bands became apparent. To identify the SOD isoforms, the gels were pre-incubated with either 1 mM KCN or 5 mM H_2_O_2_ before incubation with the reaction mixture.

### 2.4. Cloning AsSODs

Total RNA isolated from heat-treated garlic leaves was reverse-transcribed using a cDNA synthesis kit (TOYOBO Co., Ltd., Osaka, Japan). The full-length *AsSOD* genes were amplified using gene-specific primers (Table S1) designed on the basis of the transcriptome database of garlic (SRR12720215). The PCR-amplified products were cloned into the pENTR/D-TOPO vector and sub-cloned into a gateway binary vector pGWB505. The sequences of the four AsSOD genes were deposited in the National Agricultural Biotechnology Information Center (NABIC, http://nabic.rda.go.kr). Phylogenetic tree and the presence of the characteristic conserved domains in AsSODs were analyzed using Phylogeny.fr (http://www.phylogeny.fr/) and the SMART tool (http://smart.embl-heidelberg.de/), respectively.

### 2.5. Gene Expression Analysis using Quantitative Real-Time PCR (qRT-PCR)

qRT-PCR was performed using the CFX96 Real-Time PCR System (Bio-Rad, Hercules, CA, USA) with SYBR Green. The Real-time PCR data were analyzed by CFX manager software (Bio-Rad, Hercules, CA, USA) according to default parameters, which generated the Cycle Threshold (C_T_) values for each reaction. The expression levels of each gene were normalized to the constitutive expression level of garlic actin [24]. The primer sequences used for qRT-PCR analysis are listed in Supplementary Table S1. Primer efficiency values were between 95% and 98%.

### 2.6. Transient Expression of AsSODs in Nicotiana benthamiana

Plant transient expression was performed as described before [25]. Overnight cultures of *Agrobacterium tumefaciens* GV3101 containing each over-expression construct for *AsSODs* were harvested by centrifugation at 4000× *g* and resuspended to an OD600 of 1.0 in agro-infiltration media containing 10 mM MES, 10 mM MgCl_2_, and 200 µM acetosyringone. Aliquots of *A. tumefaciens* cells containing each over-expression construct were infiltrated into the leaves of tobacco (*N. benthamiana*) using 1 mL needleless syringes. Two days after infiltration, the infiltrated leaves were harvested for the SOD activity assay. Transient expression of *AsSODs* was assessed in three biological replicates.

### 2.7. Statistical Analysis

All data are presented as means ± the standard error (SE) from at least three independent experiments. The data were subjected to one-way ANOVAs using SPSS version 25 (SPSS, Chicago, IL, USA), and the Duncan’s multiple range test (*p* < 0.05) was applied to determine the level of significance of differences between the data.

## 3. Results and Discussion

### 3.1. Physiological Response to Heat Stress in Garlic Plants

**Figure 1:**
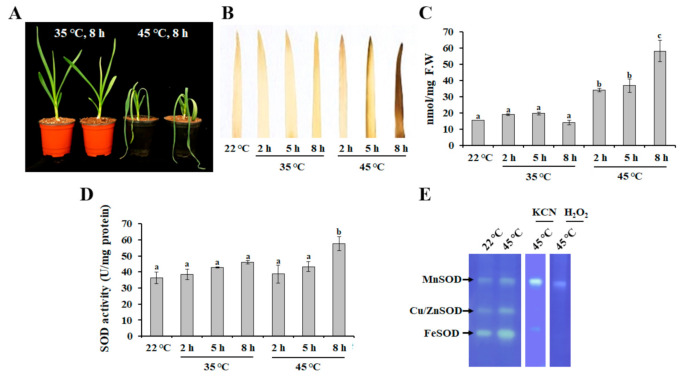
Physiological response of garlic plants under heat stress. Changes in phenotypes (**A**), H_2_O_2_ level (**B**), malondialdehyde level (**C**), total SOD activity level (**D**), and the activity pattern of SOD isozymes (**E**) were determined. In-gel SOD activity assay was performed using total protein obtained from heat-treated samples (45 °C for 8 h). The bars represent the mean ± SE across five independent experiments. Different letters indicate statistically significant differences (*p* < 0.05).

Under heat stress condition, plants exhibit various physiological and biochemical responses, such as changes in the rate of photosynthesis, growth, development, and biosynthetic pathways [26]. In addition, heat stress can disturb the balance between ROS production and antioxidant system activity [17]. Therefore, to evaluate the efficacy of heat treatment, physiological responses of garlic plants, such as lipid peroxidation and ROS accumulation, were determined. As shown in Figure 1A, leaf wilting was observed after exposure to heat stress at 45 °C for 8 h, but not at 35 °C for 8 h. Similarly, plants exposed to heat stress at 45 °C exhibited the accumulation of H_2_O_2_ (Figure 1B) and MDA (Figure 1C), which is a stable end product of lipid peroxidation [27], indicating that 45 °C is a more stressful temperature for garlic plants. In addition, a significant increase in SOD activity was also observed with increased duration of heat stress (45 °C) (Figure 1D). SOD activity has been shown to increase with an increase in temperature (until 50 °C), whereas the increasing catalase activity declined at a temperature between 30 °C and 35 °C [28,29], indicating that the accumulation of H_2_O_2_ in garlic plants exposed to heat stress at 45 °C was mediated by the decreasing activity of catalases (Figure S1). To investigate the effect of heat stress on the activity of SOD isozymes, AsSOD isozymes were separated by native PAGE gel electrophoresis and incubated with the selective inhibitors KCN (inhibitor of Cu/ZnSOD) and H_2_O_2_ (inhibitor of FeSOD and Cu/ZnSOD). As shown in Figure 1E, heat stress strongly induced the activities of Cu/ZnSODs, FeSODs, and MnSODs in leaves of garlic. Heat-tolerant varieties of *Lens culinaris* exhibited the ability to maintain increasing SOD activity at higher temperatures in comparison with heat-susceptible varieties [29], indicating that investigation of the activation patterns of SODs is required for the identification of heat-tolerant garlic varieties.

### 3.2. Cloning of SODs from Heat-Treated Garlic Plants

**Figure 2:**
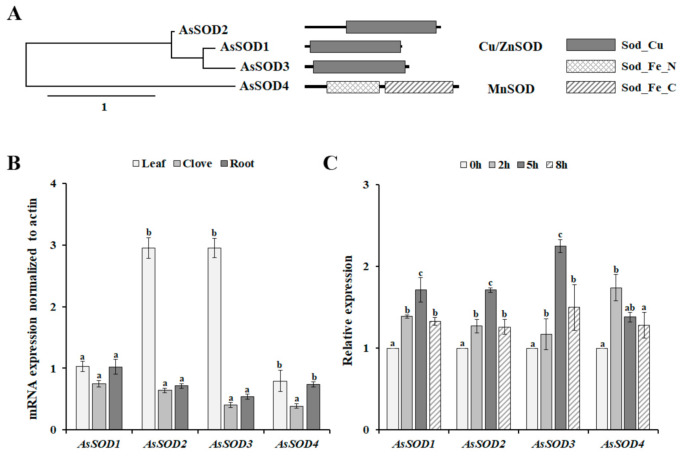
Phylogenetic tree, domain architectures (**A**), and expression patterns of putative garlic *SODs* (*AsSODs*) in different organs (**B**) and in response to heat stress (**C**). Scale bar in phylogenetic tree represents distance scale. For organ-specific expression analysis, the expression level of each gene was normalized relative to actin, whereas the normalized expression levels of *AsSODs* were expressed relative to their value at 0 h for analysis of the heat-induced expression pattern. The bars represent the mean ± SE across five independent experiments. Different letters indicate statistically significant differences (*p* < 0.05).

In the transcriptome database of garlic (SRR12720215), we found four full-length SOD sequences, together with four fragment sequences encoding different SOD proteins. Based on full-length SOD sequences, we cloned the full-length cDNAs of four putative *AsSODs*. The cloned *AsSOD* genes have an open reading frame from 459 to 711 bp, encoding 152 to 236 amino acids (Supplementary Table S2). In addition, these AsSODs have calculated molecular masses ranging from 15.28 to 26.44 kDa and theoretical pI values ranging from 5.59 to 7.10 (Table S2). In higher plants, Cu/ZnSODs are localized in all cellular compartments, whereas MnSOD is the essential enzyme that protects the energy-generating mitochondria from ROS [30,31]. Similarly, AsSOD1–3 were predicted to be cytoplasmic or chloroplast proteins, whereas AsSOD4 was predicted to be a mitochondrial protein (Table S2). The proteins belonging to the Cu/ZnSOD family are defined by the presence of the Sod_Cu domain (PF00080) [4], suggesting that three AsSODs (AsSOD1, AsSOD2, and AsSOD3) belong to the Cu/ZnSOD family (Figure 2A). The MnSOD family consists of AsSOD4, which contains two conserved domains, an iron/manganese superoxide dismutase N-terminal domain (Sod_Fe_N, PF00081), and an iron/manganese superoxide dismutase C-terminal domain (Sod_Fe_C, PF02777), which are typical of the MnSOD family [4]. Taken together, the presence of conserved domains described for each family in various plants suggests that the four cloned AsSODs belong to the SOD family.

### 3.3. Expression Patterns of the AsSOD Genes in Various Organs and in Response to Heat Stress

The analysis of organ-specific expression patterns is useful for determining whether a gene of interest plays a role in defining the function of given organs. Thus, the expression patterns of the four *AsSOD* genes were analyzed in different organs, including leaves, cloves, and roots. As shown in Figure 2B, all *AsSODs* were expressed in all tested organs. *AsSOD2* and *AsSOD3* were expressed predominantly in leaves, and their expression levels were more than 4.6 and 7.2 times higher, respectively, than those in the cloves. In contrast, *AsSOD1* and *AsSOD4* showed non-organ-specific expression patterns. Similarly, non-organ-specific expression of several SODs has been determined in cucumber, *Zostera marina*, and grapevine [4,6,31]. To analyze whether the increased SOD activity under heat stress conditions was reflected by the induction of *AsSODs*, the transcription level of each *AsSOD* in response to heat stress was analyzed using qRT-PCR. The exposure of garlic plants to heat stress induced the transcription of all tested AsSODs. The maximum transcription levels of garlic *Cu/ZnSODs* (*AsSOD1*, *AsSOD2*, and *AsSOD3*) were detected 5 h after heat-stress treatment and then declined (Figure 2C). In addition, the accumulation of garlic *MnSOD* (*AsSOD4*) expression was fast and transient and reached the highest value 2 h after heat-stress treatment. In higher plants, most *SOD* genes are induced by environmental stresses and hormones, although different expression patterns of *SODs* have been observed under diverse stresses [6,8,31]. Interestingly, heat stress leads to the downregulation of the expression of *Z*. *marina SODs* [4], whereas *AsSODs 1*–*4* are heat-inducible genes (Figure 2C), indicating the different expression patterns of *SODs* among plants. Nevertheless, this result indicates that the observed increase in garlic SOD activity (Figure 1D) is reflected by increased transcription of these *AsSODs* under heat-stress condition (Figure 2C).

### 3.4. Functional Characterization of the Four Putative AsSODs

**Figure 3:**
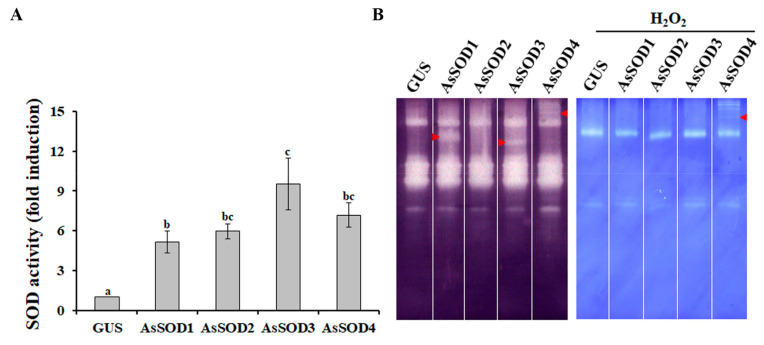
Functional characterization of putative garlic SODs (AsSODs) using the agro-infiltration assay. Total SOD activity (**A**) and activity pattern of SOD isozymes (**B**) were determined in *N*. *benthamiana* leaves infiltrated with *Agrobacterium* containing either each *AsSOD* overexpression construct or a *GUS*-overexpression construct (treated control). Arrows indicate a distinctive SOD activity band. The bars represent the mean ± SE across three independent experiments. Different letters indicate significant differences (*p* < 0.05).

Agro-infiltration is a prominent method of delivering transgenes into plant cells for the analysis of promoter activity, subcellular localization, protein function, and protein–protein interactions [32]. To analyze the functions of the four putative AsSODs, the *AsSOD* genes under the control of the CaMV35S promoter were transiently expressed in the leaves of *N. benthamiana* by using agro-infiltration. Two days after agro-infiltration, total proteins were extracted, and alterations in SOD activities were analyzed. As shown in Figure 3A, total SOD activity increased up to 9.5-fold in *N. benthamiana* leaves infiltrated with each putative AsSOD, indicating that the increased SOD activity was mediated by the expression of each *AsSOD*. In AsSOD-overexpressing leaves, distinctive SOD activity bands were also observed in native gels depending on the AsSODs (Figure 3B). Distinctive SOD activity bands obtained by overexpression of *AsSOD1*, *AsSOD3*, or *AsSOD4*, but not by overexpression of *AsSOD2*, disappeared after incubation with H_2_O_2_. *AsSOD2*-overexpressing leaves exhibited similar SOD isoform patterns as GUS-overexpressing leaves, although the total SOD activity was significantly increased (approximately 6-fold) by *AsSOD2* overexpression. This might be due to the overlap of AsSOD2 with some *N. benthamiana* Cu/Zn SOD isoforms, which can be inhibited by H_2_O_2_. Taken together, these findings indicate that *AsSOD1*, *AsSOD2*, and *AsSOD3* encode functional Cu/ZnSOD enzymes, whereas *AsSOD4* encodes an MnSOD enzyme.

## 4. Conclusions

Despite the existing knowledge concerning SODs, data for the identification and characterization of the SOD family in garlic plants are relatively scarce. In this study, we cloned and identified putative heat stress-induced *AsSOD* genes (*AsSOD1* to *AsSOD4*) belonging to the Cu/ZnSOD and MnSOD families. Using the agro-infiltration assay, we demonstrated that heat stress-induced *AsSODs* encode functional SOD enzymes. Our results provide an important starting point for future efforts to understand the function of garlic SODs in response to heat stress and provide insights for breeding programs aiming to increase the tolerance of garlic plants to global warming.

## Data Availability

The data presented in this study are available on request from the corresponding author. The data are not publicly available due to reasons of privacy.

## Supplementary Materials

The following are available online at https://www.mdpi.com/article/10.3390/antiox10050815/s1, Figure S1: Heat stress reduced total catalase activity, Table S1: Primer sequences used in this study, Table S2: Information regarding the garlic *SOD* genes cloned in this study.

## References

[1] Hasanuzzaman M., Bhuyan M.H.M., Zulfiqar F., Raza A., Mohsin S.M., Mahmud J.A., Fujita M., Fotopoulos V. (2020). Reactive oxygen species and antioxidant defense in plants under abiotic stress: Revisiting the crucial role of a universal defense regulator. Antioxidants.

[2] Huang H., Ullah F., Zhou D.X., Yi M., Zhao Y. (2019). Mechanisms of ROS regulation of plant development and stress responses. Front. Plant Sci..

[3] Mittler R. (2017). ROS are good. Trends Plant Sci..

[4] Zang Y., Chen J., Li R., Shang S., Tang X. (2020). Genome-wide analysis of the superoxide dismutase (SOD) gene family in *Zostera marina* and expression profile analysis under temperature stress. PeerJ.

[5] Kliebenstein D.J., Monde R.A., Last R.L. (1998). Superoxide dismutase in Arabidopsis: An eclectic enzyme family with disparate regulation and protein localization. Plant Physiol..

[6] Hu X., Hao C., Cheng Z.M., Zhong Y. (2019). Genome-wide identification, characterization, and expression analysis of the grapevine superoxide dismutase (SOD) family. Int. J. Genom..

[7] Verma D., Lakhanpal N., Singh K. (2019). Genome-wide identification and characterization of abiotic-stress responsive SOD (superoxide dismutase) gene family in *Brassica juncea* and *B. rapa*. BMC Genom..

[8] Zhou C., Zhu C., Fu H., Li X., Chen L., Lin Y., Lai Z., Guo Y. (2019). Genome-wide investigation of superoxide dismutase (SOD) gene family and their regulatory miRNAs reveal the involvement in abiotic stress and hormone response in tea plant (*Camellia sinensis*). PLoS ONE.

[9] Jiang W., Yang L., He Y., Zhang H., Li W., Chen H., Ma D., Yin J. (2019). Genome-wide identification and transcriptional expression analysis of superoxide dismutase (SOD) family in wheat (*Triticum aestivum*). PeerJ.

[10] Han L.M., Hua W.P., Cao X.Y., Yan J.A., Chen C., Wang Z.Z. (2020). Genome-wide identification and expression analysis of the superoxide dismutase (SOD) gene family in *Salvia miltiorrhiza*. Gene.

[11] Borrego-Benjumea A., Carter A., Tucker J.R., Yao Z., Xu W., Badea A. (2020). Genome-wide analysis of gene expression provides new insights into waterlogging responses in barley (*Hordeum vulgare* L.). Plants.

[12] Lu W., Duanmu H., Qiao Y., Jin X., Yu Y., Yu L., Chen C. (2020). Genome-wide identification and characterization of the soybean SOD family during alkaline stress. PeerJ.

[13] Guan Q., Liao X., He M., Li X., Wang Z., Ma H., Yu S., Liu S. (2017). Tolerance analysis of chloroplast OsCu/Zn-SOD overexpressing rice under NaCl and NaHCO_3_ stress. PLoS ONE.

[14] Rubio M.C., González E.M., Minchin F.R., Webb K.J., Arrese-Igor C., Ramos J., Becana M. (2002). Effects of water stress on antioxidant enzymes of leaves and nodules of transgenic alfalfa overexpressing superoxide dismutases. Physiol. Plant..

[15] Tseng M.J., Liu C.W., Yiu J.C. (2007). Enhanced tolerance to sulfur dioxide and salt stress of transgenic Chinese cabbage plants expressing both superoxide dismutase and catalase in chloroplasts. Plant Physiol. Biochem..

[16] Prashanth S.R., Sadhasivam V., Parida A. (2008). Over expression of cytosolic copper/zinc superoxide dismutase from a mangrove plant *Avicennia marina* in indica rice var Pusa Basmati-1 confers abiotic stress tolerance. Transgenic Res..

[17] Chalanika De Silva H.C., Asaeda T. (2017). Effects of heat stress on growth, photosynthetic pigments, oxidative damage and competitive capacity of three submerged macrophytes. J. Plant Interact..

[18] Kumar N., Ebel R.C., Roberts P.D. (2011). Antioxidant isozyme variability in different genotypes of citrus and kumquat. J. Crop Improv..

[19] Berwal M.K., Sugatha P., Niral V., Hebbar K.B. (2016). Variability in superoxide dismutase isoforms in tall and dwarf cultivars of coconut (*Cocos nucifera* L.) leaves. Indian J Agric. Biochem..

[20] Chen X., Liu X., Zhu S., Tang S., Mei S., Chen J., Li S., Liu M., Gu Y., Dai Q. (2018). Transcriptome-referenced association study of clove shape traits in garlic. DNA Res..

[21] Daudi A., O’Brien J.A. (2012). Detection of hydrogen peroxide by dab staining in Arabidopsis leaves. Bio Protoc..

[22] Eom S.H., Hyun T.K. (2021). Comprehensive analysis of the histone deacetylase gene family in Chinese cabbage (*Brassica rapa*): From evolution and expression pattern to functional analysis of BraHDA3. Agriculture.

[23] Beyer W.F., Fridovich I. (1987). Assaying for superoxide dismutase activity: Some large consequences of minor changes in conditions. Anal. Biochem..

[24] Wang G., Tian C., Wang Y., Wan F., Hu L., Xiong A., Tian J. (2019). Selection of reliable reference genes for quantitative RT-PCR in garlic under salt stress. PeerJ.

[25] Hyun T.K., van der Graaff E., Albacete A., Eom S.H., Großkinsky D.K., Böhm H., Janschek U., Rim Y., Ali W.W., Kim S.Y. (2014). The Arabidopsis PLAT domain protein1 is critically involved in abiotic stress tolerance. PLoS ONE.

[26] Hassan M.U., Chattha M.U., Khan I., Chattha M.B., Aslam M.T. (2020). Heat stress in cultivated plants: Nature, impact, mechanisms, and mitigation strategies—A review. Plant Biosyst..

[27] Morales M., Munné-Bosch S. (2019). Malondialdehyde: Facts and artifacts. Plant Physiol..

[28] Gupta S., Gupta N.K. (2005). High temperature-induced antioxidative defense mechanism in seedlings of contrasting wheat genotypes. Indian J. Plant Physiol..

[29] Chakraborty U., Pradhan D. (2011). High temperature-induced oxidative stress in *Lens culinaris*, role of antioxidants and amelioration of stress by chemical pre-treatments. J. Plant Interact..

[30] Huseynova I.M., Aliyeva D.R., Aliyev J.A. (2014). Subcellular localization and responses of superoxide dismutase isoforms in local wheat varieties subjected to continuous soil drought. Plant Physiol. Biochem..

[31] Zhou Y., Hu L., Wu H., Jiang L., Liu S. (2017). Genome-wide identification and transcriptional expression analysis of cucumber superoxide dismutase (SOD) family in response to various abiotic stresses. Int. J. Genom..

[32] Norkunas K., Harding R., Dale J., Dugdale B. (2018). Improving agroinfiltration-based transient gene expression in *Nicotiana benthamiana*. Plant Methods.

