# Supplementary material for: Molecular Cloning and Functional Characterization of Heat Stress-Responsive Superoxide Dismutases in Garlic (Allium sativum L.)
Source: Antioxidants (Basel). 2021 May 20;10(5):815. doi: 10.3390/antiox10050815 (PMC8161062; doi:10.3390/antiox10050815)
Supplement: Supplementary file 1 [file antioxidants-10-00815-s001.zip › antioxidants-1217270-supplementary.pdf]

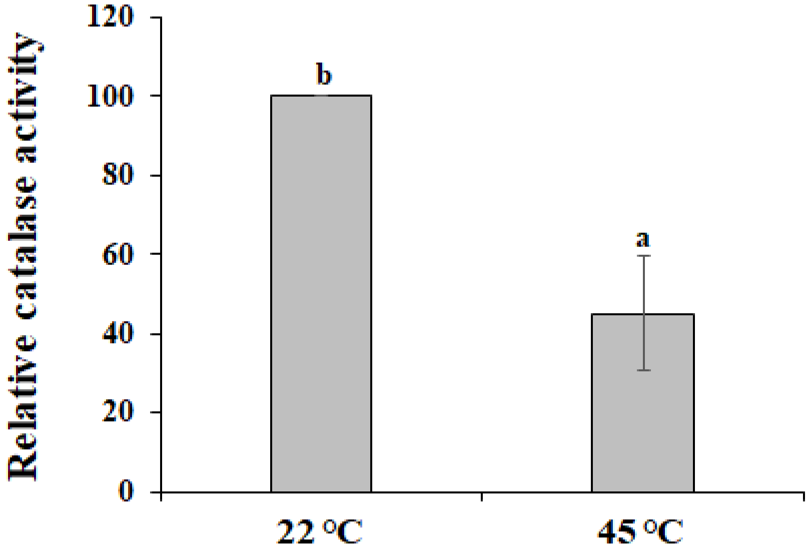

Figure S1. Heat stress reduced total catalase activity.

Table S1. Primer sequences used in this study.

	Primer	Sequences (5'–3')	Accession number/Reference
For cloning	AS_SOD1-F	CACCATGGTGAAGGCAGTTACTG	NS-3057
	AS_SOD1-Rev	GCTCTGGAGTCCAATGATACC	
	AS_SOD2-F	CACCATGGCACTCAGAGCACTC	NS-3058
	AS_SOD2-Rev	CCAAGAGTAGCTCGTGCAAAT	
	AS_SOD3-F	CACCATGGCTTCTTCAATCGCT	NS-3059
	AS_SOD3-Rev	TGCCACTGGTGTCAATCCAAC	
	AS_SOD4-F	CACCATGTCTTTCTTCCGACTC	NS-3060
	AS_SOD4-Rev	TGCAGTCTCTTTTTCATACAC	
For qRT-PCR	AsSOD1-F	GCCATGCTGGTGACCTTGGA	NS-3057
	AsSOD1-Rev	GGGCCAGTGAGTGGGATCTG	
	AsSOD2-F	GGCGAAGCAAACAGCCTCAT	NS-3058
	AsSOD2-Rev	AGTATCGCCGAACGAGTGGA	
	AsSOD3-F	TGGAGTGGCAGAGGCGACTA	NS-3059
	AsSOD3-Rev	TCCCAGTGCTGAGGCTGAGT	
	AsSOD4-F	GGCGAGCCTCCTCATGGTTC	NS-3060
	AsSOD4-Rev	CCAGAGCAAGCCACACCCAA	
	AsACTIN-F	TGCTCTGGATTATGAACAGGAACTTGA	Wang et al. 2019 [24]
	AsACTIN-Rev	CAATCATTGAAGGCTGGAACAACACT	

Table S2. Information of garlic *SOD* genes cloned in this study.

Name	CDS (bp)	AA	pI	kDa	Localization [a]	Accession number (NABIC)
AsSOD1	459	152	5.59	15.28	Cytoplasm	NS-3057
AsSOD2	492	163	6.28	16.73	Cytoplasm	NS-3058
AsSOD3	633	210	6.74	21.36	Chloroplast	NS-3059
AsSOD4	711	236	7.10	26.44	Mitochondrion	NS-3060

[a] Prediction by Plant-mSubP.
